# Men’s mental health and the arts: perceived benefits and dynamics of engagement

**DOI:** 10.1093/heapro/daad092

**Published:** 2023-08-17

**Authors:** Shane O’Donnell, Maria Lohan, John L Oliffe, David Grant, Noel Richardson, Karen Galway

**Affiliations:** Department of Health and Sports Sciences, The National Centre for Men’s Health, South East Technological University, Kilkenny Road Campus, Kilkenny Road, Carlow R93 V960, Ireland; School of Nursing and Midwifery, Queen’s University Belfast, Medical Biology Centre, 97 Lisburn Road, Belfast BT9 7BL, UK; School of Nursing, University of British Columbia, 302-6190 Agronomy Road, Vancouver, British Columbia V6T 1Z3, Canada; Department of Nursing, University of Melbourne, Grattan Street, Parkville, Victoria 3010, Australia; School of Arts, English and Languages, Queen’s University Belfast, 2 University Square, Belfast BT7 1NN, UK; Department of Health and Sports Sciences, The National Centre for Men’s Health, South East Technological University, Kilkenny Road Campus, Kilkenny Road, Carlow R93 V960, Ireland; School of Nursing and Midwifery, Queen’s University Belfast, Medical Biology Centre, 97 Lisburn Road, Belfast BT9 7BL, UK

**Keywords:** arts, men, mental health, engagement, masculinity

## Abstract

Arts engagement is gaining recognition as a non-clinical approach to promote mental health and well-being. However, the perceived utility of the arts to promote mental health among men with low socioeconomic status (SES) and how to best engage them is underexplored. This study explores the lived experiences of men with low SES who engage with the arts in Northern Ireland (*n* = 41). Data collected via focus groups (*n* = 5) and interviews (*n* = 11) were analysed using reflexive thematic analysis to inductively derive four themes. Theme 1 highlights how the arts facilitated friendship, a collective identity, peer support and a reason to socialize. Themes 2 and 3 explore how the arts enhanced self-esteem and emotional regulation by developing a routine, purpose, sense of mastery, a sense of catharsis through immersion in a soothing endeavour and an alternative outlet for self-expression. Theme 4 covers strategies that facilitate male engagement in the arts such as using a familiar space, delivering to an existing male group, framing the programme around male interests not health or creativity, building on existing strengths and capacities, enabling ownership, using tangible action-orientated activities, and being non-authoritative and flexible with delivery. This is one of the first studies to highlight the gendered dimensions in which men with low SES engage with and experience mental health benefits through arts engagement. This study points towards relevant theories to further understand the pathways between the arts and improved mental health among men which can inform development of tailored arts programmes for men.

**Contribution to Health Promotion**

- Highlights how the arts positively impact the mental health of men from economically deprived communities through enhanced social connections, self-esteem and emotional regulation.
- Highlights strategies to engage men from economically deprived communities in the arts.
- Builds the credibility of using the arts to promote mental health among men from economically deprived communities.
- Advances theories to conceptualize the evidence base of arts engagement for improving mental health among men from economically deprived communities.

## BACKGROUND

Men’s mental health challenges have been labelled as a ‘silent epidemic’ (Whitley, 2018). Common mental health issues including anxiety and depression are considerable contributors to the global burden of disease among males (Baker *et al*., 2014; Rehm and Shield, 2019). The extent of this ‘silent epidemic’ is highlighted by the male suicide rate which is three times greater than the female rate in high-income countries (WHO, 2021). Many studies have utilized Connell’s social constructionist theory on masculinities to explore the relationship between men and mental health challenges (Connell and Messerschmidt, 2005; Scourfield, 2005; Oliffe *et al*., 2017; Apesoa-Varano *et al*., 2018; O’Donnell and Richardson, 2020). Conformity to ‘dominant’ masculine standards such as self-reliance, control and concealing vulnerability are implicated due to their association with men’s reticence to seek help for mental health challenges (Galdas *et al*., 2005; Siedler *et al*., 2016; McDermott *et al*., 2018). In addition, barriers to male engagement with health are linked to the social construction of masculinities within the context of broader intersectoral inequalities in health (Lohan, 2007). This is compounded by structural barriers whereby services are reported to be insufficiently sensitive to the connections between masculine ideals and men’s reticence for mental health help-seeking (Brownhill *et al*., 2003; Seidler *et al*., 2021). Therefore, engaging men around their mental health requires navigating these diverse masculine norms through incorporating gender-responsive approaches into tailored interventions (Struik *et al*., 2019). This has prompted the development of a range of non-clinical programmes which have mostly been confined to workplace interventions (Gullestrup *et al*., 2011; Johnson *et al*., 2016), exercise-based interventions (McGale *et al*., 2011; Sharp *et al*., 2021) and social-based interventions (Fildes *et al*., 2010; McGrath *et al*., 2022). Such interventions have positively impacted men’s perceptions of mental health through the purposeful use of men’s interests, team-based activities and/or familiar spaces to facilitate the delivery of mental health messages, harness social connection and normalize mental health and help-seeking (Gullestrup *et al*., 2011; Sharp *et al*., 2021; McGrath *et al*., 2022). There is also a growing evidence base for the role of arts engagement to promote mental health and well-being in non-clinical settings (Fancourt and Finn, 2019); yet its utility to promote mental health among men is underexplored.

Defining the arts can be conceptually difficult (Adajian, 2018) but several cross-cutting characteristics are proposed as fundamental to what constitutes ‘art’: a physical or experiential art object that is valued in its own right; an imaginative experience for the producer or audience; comprising or provoking an emotional response; requiring creativity or originality; and specialized skills that relate to the rules of the form, composition or expression (Fancourt and Finn, 2019). Within this frame arts engagement has been categorized as ‘active’ or ‘passive’ engagement in the following five forms: (i) performing arts; (ii) visual arts; (ii) design and craft; (iv) literature, community and cultural festivals; and (v) online, digital and electronic arts (Davies *et al*., 2012). This definition of arts engagement forms the basis of the current article. While it is acknowledged that this definition is built on historical assumptions that value particular forms of activities over others that may overlook the value of everyday activities such as gardening or cooking (Miles and Gibson, 2016), a focus on wider concepts of creativity, cultural and everyday participation resides outside the purpose of the current research and article.

Arts engagement often constitutes the invoking of emotions, aesthetic engagement, imagination, sensory activation, cognitive stimulation, social connection, physical exertion and/or engagement with health topics (Fancourt, 2017). Therefore, these components of arts engagement can positively influence mental health via physiological, psychological, social and/or behavioural pathways. Indeed, recent systematic reviews have reported the positive impact of arts engagement on social cohesion, confidence and self-esteem, empowerment, self-expression and identity re-formation (Stickley *et al*., 2018; Sheppard and Broughton, 2020; O’Donnell *et al*., 2022). Arts engagement has also been found to reduce symptoms associated with depression and anxiety (Fancourt *et al*., 2016; Williams *et al*., 2018), aid recovery among people experiencing common mental health problems (Gallant *et al*., 2019; Hui *et al*., 2019) and improve mental well-being (Crone *et al*., 2013; Fancourt and Finn, 2019). Despite this growing evidence base in support of arts engagement and health gains in non-clinical settings, there is a dearth of research on the mental health impact of arts engagement among men, particularly men of low socioeconomic status (SES). A small number of studies reported improved mental well-being, social relationships, a sense of achievement, routine and structure among men following arts engagement (Thomas *et al*., 2011; Heard *et al*., 2013; Wilkinson and Caulfield, 2017). However, these studies did not set out to explicitly recruit men or explore men’s experiences of the arts and were not underpinned by social constructionist theories of masculinities. As such, there remains a lack of understanding in the wider literature with regard to the gendered factors that influence relationship between arts engagement and improved mental health among men (O’Donnell *et al*., 2022). Moreover, the lack of research on arts engagement among men of low SES is likely due to the underrepresentation of people of low SES and men in the arts more generally (Lehman and Dumais, 2017; Fancourt and Mak, 2020; Mak *et al*., 2020). Some studies have proposed that these demographic groups have fewer opportunities for arts engagement, view the arts as a feminine activity and commonly experience more mental health difficulties, all of which affects their motivation to participate (Christin, 2012; Lagaert *et al*., 2017; Fancourt and Mak, 2020). However, more research is needed to explore the barriers and enablers to arts engagement specifically among men of low SES. Indeed, similar to men’s engagement with health services, lack of participation may not denote low interest or motivation but could be influenced by wider attitudinal and structural barriers that are in turn malleable with gender-responsive approaches. Therefore, this study sought to gain a deeper understanding of the gendered experiences of arts engagement among men of low SES through the following two research questions:

1. What are the perceived mental health impacts of arts engagement among men of low SES?
2. What were the barriers and enablers to arts engagement among men of low SES?

## METHODS

Ethical approval was granted from the School of Nursing and Midwifery research ethics committee at Queen’s University Belfast (Reference: 1.SO’Donnell 09.18.m1.V2). Individuals were eligible to participate if: they self-identified as male; were ≥18 years old; were engaged in ‘the arts’ within an all-male group; and resided in an area of low SES in Northern Ireland. The arts were defined in line with the definition previously highlighted (Davies *et al*., 2012). An area of low SES was defined as a geographical community that ranked within the 20% most deprived communities of Northern Ireland as defined by the Northern Ireland Multiple Deprivation Index (NISRA, 2017). Participants were recruited using purposive and snowball sampling techniques. Gatekeepers in relevant community and voluntary organizations within the 20% most deprived communities in Northern Ireland were identified through online searching and the networks of the authors. Gatekeepers were contacted to explain the purpose of the study and were asked to support recruitment. Gatekeepers identified eight relevant groups. They were contacted by the lead researcher via phone to discuss their eligibility and willingness to participate. Six groups agreed to participate, one did not respond and an appropriate time could not be agreed with the remaining group. The first author visited the six men’s groups to explain the purpose of the research, circulate information sheets and allow men to ask questions. Those who wished to participate gave their contact details to the lead author via email and/or phone and a mutually convenient time was agreed to conduct the focus group. Some men wished to participate but did not want to discuss their experiences in front of their peers. These men were offered a semi-structured individual interview as an alternative data collection method.

Prior to data collection, the lead author reiterated the purpose of the study and explained that participation was voluntary before obtaining written consent. The topic guide used open-ended questions to explore the mental health impact of the arts and the factors that facilitated or impeded their engagement within the context of an all-male group. The interview questions were purposefully open-ended to encourage participants to freely self-define what they considered to be arts engagement. Therefore, participants defined the arts based on their own experiences and they discussed various art forms in response to interview questions including ‘What are your experiences of engaging with art in this group?’. Participants were directly asked about their experiences of arts engagement within the group setting but inevitably, some men also spoke to their broader experiences of arts engagement outside the group. Participants were provided with contact details for support services and encouraged to access them if issues arose in taking part in the research. Data collection ceased once no new information was observed during data collection. Five focus groups and 11 individual interviews were audio-recorded which lasted on average 46–56 min. All data were collected by the lead author from May to November 2019, anonymized, cleaned of any identifying information and pseudonyms generated for each participant.

As this study was concerned with exploring men’s gendered experiences of arts engagement, the authors understand gender and masculinities as a social construct (Connell, 2005). Therefore, our research was situated within a social constructionist approach that acknowledges the influence of social, cultural and historical contexts on doing gender and masculinities. The interpretation of the data was grounded in Connell’s (Connell, 2005) social constructionist theory on masculinities. As such, reflexive thematic analysis was deemed most appropriate to examine men’s lived experiences of arts engagement, whilst also identifying the social processes that shape these experiences, meanings and assumptions (Braun and Clarke, 2013). Braun and Clarke’s six-step approach to reflexive thematic analysis was utilized to interpret the data (Braun and Clarke, 2021). The focus groups and interviews were transcribed verbatim by the lead author, transcripts were read multiple times, and initial observations on patterns within and across transcripts were recorded to ensure data familiarization and immersion. Guided by the research questions, an inductive approach to coding was adopted wherein initial codes were generated for each individual transcript that looked at the semantic and latent level to explore the ways that men described doing gender within the context of arts engagement. These codes were discussed and revisited on several occasions by the authors as the analysis progressed to consider assumptions held by the research team, how the data could be interpreted differently and potential deeper meanings behind the data. Codes were grouped into similar patterns to form preliminary themes using tables and conceptual maps to track iterations of, and relationships between, codes and patterns. The authors discussed and worked with the data sets to refine themes by ensuring that the associated data was meaningful and coherent within themes and that there were identifiable differences across themes. This process resulted in revised codes, patterns and themes. The refined themes were then re-read against the transcripts to ensure they were consistent with the entire data set. Finally, agreement was reached about theme names and content upon which analysis were built and finalized in the writing up of this article. Field notes and a reflexive journal were used to record observations, assumptions and to contextualize these verbal accounts during transcription and data analysis.

## RESULTS

A total of 41 men participated in this study with an average age of 63.7 years old (SD 13.3 years) who were mostly White Irish/British/Northern Irish (*n* = 40; 97.5%). The majority of participants were either retired (*n* = 20; 48.7%), away from work due to illness (*n* = 9; 22%) or unemployed (*n* = 9; 22%). Half the participants were not married (*n* = 21; 51%) and just under two-fifths lived alone (*n* = 16; 39%). Participants resided in communities in the top 0.35–12.4 percentile of deprivation in Northern Ireland. Of the six groups that participated—four were men’s sheds group and two were mental health recovery groups. Therefore, participants did not explicitly seek out arts engagement but found an interest in the arts through exposure in their respective groups. The primary reason for participants’ initial engagement with these groups was mental ill-health, social connection, encouragement from family and/or the group seeking new members. Finally, all these groups adopted a peer-led model so arts engagement was dependent on collective interests and opportunities available to the group. There were no instances of passive engagement and the most common forms of active engagement related to craft-making, woodturning, learning musical instruments, choir singing and drawing followed by drama, painting and photography. There were no observable differences in the data and initial codes emerging from the focus groups and individual interviews. Therefore, the data sets were combined and the findings drawn are presented as four overarching themes: (i) social connectedness, (ii) self-esteem, (iii) emotional regulation and (iv) the dynamics of male engagement in the arts.

### Theme 1: Social connectedness

Social connectedness was the most commonly reported benefit of engaging in the arts. Most participants reported flourishing friendships, a sense of belonging and acceptance—a trifecta of benefits accrued from engaging with their respective groups.

*I made some friends which was nice. When you make a friend that is quite lovely, that was one of the real positives of coming here.* (Joey, 53, Focus Group 5)

*See as soon as you walk through that door? You are accepted … something I never had before.* (Paddy, 45, Focus Group 1)

*It was great friendship, then every Saturday they started to come to my house and play [music].* (Bob, 58 Interview)

A shared interest in the arts was a focal point around which friendships were formed. This shared interest also facilitated socializing together outside group time which further strengthened a sense of connectedness. The arts also represented an action-orientated activity that facilitated a comfortability for men to discuss mental health. Holding informal conversation whilst engaging in arts activities appeared to lessen the sense of vulnerabilities for discussing mental health challenges wherein such conversations were by-products of an activity rather than a direct focus (and pressures to talk).

*These sort of things [the arts] are coming at mental health from a different angle and that works better for men I think. You are here making something with somebody and then you would get into a conversation about mental health or something. There is a lot of jest and joking in it too but that is the way we talk in here.* (Tim, 52, Interview)

This focus on ‘doing’ ultimately formed the foundations for an environment conducive to mental health peer support where men felt comfortable opening up to peers.

*Before I came here I kept everything in. I was getting worse and worse. Men don’t talk but in here you can talk about anything. You know it doesn’t go outside that door.* (Peter, 41, Focus Group 2)

Participants noted that humour, ‘banter’ and ‘slagging’ (slang for teasing in a light-hearted manner) were key modes of male bonding and significantly contributed to a sense of group camaraderie. This informal chat was also felt to be supportive in terms of providing a relief and distraction from ongoing mental health struggles (‘*we talk rubbish but the rubbish helps’ Oscar, 64*). Working towards a shared goal and an art ‘product’ helped to create a collective identity among the group and contributed to a sense of belonging for individuals. Bob noted how the physical synchrony of collective creativity uplifted his mood and facilitated a sense of connectedness.

We all work together … we are all playing together. I play along with him and I know he is coming in next. When we get it right and everything goes right and the end bit comes and you think f**k that was good, you get a buzz. We look at each other and smile. (Bob, 58, Interview)

Although the arts helped to drive connectedness within the group, it also acted as a medium to establish connectedness to their wider communities. Sam highlighted how the arts helped to reconnect him to a world in which he felt he had become a spectator since his retirement.

*When you are retired … the world is going on and going past you—you are not involved in it. But things like this [participatory arts] bring you into that world, it keeps you in touch with everything that’s going on. You are involved with making benches for schools … singing in nursing homes … you are out there.* (Sam, 65, Focus Group 3)

### Theme 2: Self-esteem

Men reported that the arts contributed to positive transformations in their sense of purpose, identity and overall self-esteem by offering routine and meaningful work, eliciting a sense of achievement and self-confidence in their abilities, providing opportunities for altruism and active citizenship, and sharing their knowledge and skills with others. Engagement in the arts was felt to be a meaningful activity that was constructive, positive and a worthwhile use of time. This helped to re-ignite a sense of routine and purpose, meaningful contribution—elements which were often lost to retirement or the absence of paid work.

*This is where the creativity stuff comes in—to feel useful again. It keeps me occupied. Something happens in your life, that you get down and you don’t feel useful to society anymore. You are in that society, going out to work … and then it kind of gets taken away from you. You are on the scrapheap, but this [the arts] helps you get out of that situation. It is somewhere to go, someone to talk to and something to do*. (Joe, 54, Focus Group 1)

The artistic process facilitated a sense of achievement through the acquisition of new skills and knowledge which also led to greater self-confidence that cascaded into other areas of life. One participant noted how his singing and poetry gave him greater self-confidence and the ability to better cope with past traumas. Tim’s quote below also highlights the power of the arts in overcoming self-doubt and perceived inadequacies associated with depression.

*Towards the end of the 6 weeks my drawings were a lot better. It’s getting rid of the ‘I can’t’. Depression says you can’t but once you get something out creatively you start to break that down*. (Tim, 52, Interview)

Moreover, the art product was viewed as a tangible outcome, the fruits of men’s labour, that personified increased competencies and represented wise investments of time, energy and dedication.

*It’s a sense of I have done this, pride in my achievements, this is what I have done, this is what I am able to do. Using the mind and putting those things into action and what we see in the fruits of our labour. Creativity lifts us up.* (Arnold, 59, Focus Group 5)

Men favoured creative endeavours that were ‘useful’ to others and that enabled them to provide value to community members (*‘its art but it has a use’ Karl, 65*). Examples of such endeavours included making Christmas decorations for the local community, benches for local schools and singing in local shopping centres and nursing homes. The act of giving was a positive experience in and of itself but the affirmations reciprocated by other men reinforced feelings of enhanced self-esteem and self-belief.

*You feel that you are useful again. That you are doing something good for the general public. We go to nursing homes and sing now and again and it is satisfying to see people singing along and smiling. It is sort of like giving back to the community.* (David, 70, Focus Group 4)

Finally, the arts enhanced self-esteem by enabling men to share their own expertise and knowledge within the group. This appeared to help men remember parts of themselves that had temporarily been lost through displaced roles and perceived inadequacies. Joey described tapping into his background in drama to assist in the development of a play, which made him feel like he was making a worthwhile contribution to his group.

*We developed a play last year. That was in my area of expertise and that helped my mental health so much. I felt like I was contributing something to the group. Taking whatever skills I have and helping others in a positive way*. (Joey, 53, Focus Group 5)

### Theme 3: Emotional regulation

Men highlighted how the arts provided them with solace through immersion in a soothing and nurturing endeavour which helped to counterbalance and distract them from past traumas. Liam highlighted the importance of the arts in distracting him from past negative experiences and likened his pursuits of visual arts and music as his ‘proper psychiatrist’.

*Art and music are really big for my mental health, I call them the proper psychiatrist. They focus your mind on one thing, if you’re doing something like music or art you are only focused on that. It is a good thing then it prevents me from focusing on all the negative stuff in my past.* (Liam, 79, Focus Group 2)

Victor also highlighted how the creative process lifted him out of a more insular and ruminating depressive state into a new mindset.

*When you suffer from depression you become insular, you are just thinking about yourself and your problems—you are always down on yourself. But whenever you are doing something creative in a group, like the choir or the art class, you are concentrating on that and everything else is gone. One of the best things we ever did was the formation of the choir.* (Victor, 72, Interview)

This concentration and focus on the task at hand was grounding whereby men became present and in the moment. In addition to providing a ‘distraction’ from mental health issues, complete focus during arts engagement was a pleasurable experience in and of itself. Many participants spoke of their enjoyment of the quietness and described an altered sense of timelessness, contemplation and of being in the present moment when engaged in the artistic process. Indeed, men described the process as relaxing, calming and therapeutic. This was particularly related to art forms that required less physical activity such as painting, drawing and calligraphy.

*It puts me in a reverie and I forget everything else*. (Billy, 79, Interview)

*It is therapeutic. There is just enjoyment in the quietness, just getting on with something you enjoy doing. You are sort of in your own world kind of a thing.* (Sean, 61, Focus Group 3)

Engaging in the arts was also seen as filling a void that might otherwise be occupied by other activities such as going to the pub. Beyond negating the impact of more challenging emotions, the arts helped to generate more uplifting emotions such as joy and happiness. Many noted that the arts provided a ‘boost’ and elicited a ‘feel good factor’ that lasted well beyond the activity ending. There seemed to be an intrinsic joy and satisfaction experienced during the act of creation and a sense of awe in seeing ideas transition through to tangible products or performances.

*The effect it has on your mental health—just creating something. It doesn’t go away, that positive feeling stays with you and you feel good about yourself. I created something.* (Victor, 72, Interview)

The arts were felt to be an outlet for self-expression which was described as cathartic and therapeutic. Arts engagement was valued as an alternative form of ‘communication’ particularly for men who found it difficult to express emotions including sadness, due to a perceived pressure to be stoic and strong.

*As men we wear masks all the time … but creative activities allow us to express ourselves in ways that we feel we are not allowed to. So that creativity allows us to take that mask off which can be very therapeutic.* (Arnold, 59, Focus Group 5)

Tim highlighted the importance of creative self-expression (‘getting things out’) in offsetting the insularity of depression (‘keeping things in’) and avoiding more unhelpful or ‘male-acceptable’ coping mechanisms. Here, he could derive meaning and depth to release his emotions and arrest the interiority of what was being negatively felt.

*What are we without art? Art is about expressing yourself and getting something out because depression wants to keep you in. It has to come out someway and it is better to get it out onto a page than drinking or rallying up the road.* (Tim, 52, Interview)

### Theme 4: Dynamics of male engagement in the arts

Many participants described their initial reticence and scepticism about engaging in the arts which they felt was a feminine hobby and ‘not for working-class men’ (Joey, 53, Focus Group 5). However, creating a safe space and a sense of trust between group members helped to alleviate concerns being emasculated by *their* arts engagement. This was facilitated through a sense of relatedness among group members, hosting the arts activities in venues that were familiar and comfortable to men and specifically advertising the programme as a male space. Daniel described how this norming of a men’s group and the ease with which opportunities were provided facilitated their arts engagement.

*If you were asked to do it yourself you would kind of be wary. But once you are in the crowd … you just engage because you are in the safety of the group. It could be any activity, it wouldn’t matter what it is, we would do it … once you do it you realise you enjoy it.* (Daniel, 46, Focus Group 1)

Beyond creating a more comfortable space, ‘doing’ normed being amongst other men rather than the passivity sitting and talking which could reinforce feelings of isolation and declining physical functioning. Therefore, men highlighted the importance of utilizing more action-orientated art activities over passive forms of engagement.

*I think what is important is having an action-orientated activity to do in the arts. I wanted the group to be active doing things and making stuff, not sitting in a wee room chatting.* (John, 63, Focus Group 1)

Participants described the encouraging and enthusiastic style of the facilitator as crucial to their engagement, an approach which piqued men’s interests without any pressure to participate. This ultimately left men feeling they had autonomy and the choice to participate.

*There is an energy there and he presents it as something you can do with your time. He would be full of encouragement. It naturally gets you interested and then when you are interested you enquire a bit more and get involved. There is no pressure there*. (Jarlath, 65, Interview)

Participants recounted how the more effective artistic facilitators carefully dispelled any perceptions of a hierarchy or power imbalance within the group. They were not being overly strict or authoritative, and by ensuring all men’s voices were heard, respected and valued. Strength-based strategies that revolved around giving men ownership over the process and prioritizing peer-led approaches in programme delivery were seen as fundamental and helped men to feel more relaxed and to be guided and moved to do things.

*You can’t say right we are going to have an art class and expect all the men to come along. You have to be non-directive with some sort of an outcome … give those men a degree of ownership as well. The play we did, there was an outline idea but the only hard outcome was to have a piece in a set time. We all had ownership to it.* (Arnold, 59, Focus Group 5)

Flexibility with participation provided a bridge for men to dabble in the arts without feeling the need to fully commit. Indeed, the option to ‘come and go as you please’ (Jarlath, 65, Interview) was a factor that attracted many men to engage in the first instance. This flexibility to dip in and out of activities was particularly important for men whose attendance was more sporadic due to experiencing mental health ill-health.

*That is one of the great things about here, if you were out of here for a few days or weeks because of depression, when you come back you don’t feel like a project is nearly complete or I am 2 or 3 weeks behind on a course. There is no kind of pressure for people coming back, you just come back in and sit on the same seat and get on with it.* (Phillip, 61, Focus Group 2)

Other common techniques deployed by artistic facilitators to overcome participants’ fear of failure and perceived lack of artistic skills was beginning with activities that had a lower skill threshold to participate and graduating to more difficult tasks. For example, one group began with tracing pictures of scenery, before moving on to portraits and more detailed tasks like shading and colouring. Finally, participants stressed the importance of producing something tangible at the end of the creative process. There was a perception that men were very much ‘outcome-based’ and that having a goal or a solution to work towards often resulted in a more positive engagement. However, in noting the importance of having an end product, it was highlighted that the quality of the product should not take precedence. Rather, there was recognition that the focus should be on the enjoyment of the creative process. This helped to alleviate concerns of a perceived lack of artistic skill and frustrations with the quality of the art.

*As men we are very outcome-based … so we like to know what we are going to make or produce before we start, you can imagine what it is you are working to … but the process is as important because it is in that process that those interactions occur. People are terrified of the arts because they think ‘I will not be very good at that’. You don’t have to be brilliant at what you do, you just have to enjoy it.* (Joey, 53, Focus Group 5)

## DISCUSSION

Arts engagement was perceived to positively contribute to the mental health of men of low SES by enhancing social connectedness, self-esteem and emotional regulation. Social capital theory (Putnam, 2000), social cognitive theory (Bandura, 1986) and the process model of emotional regulation (Gross and Thompson, 2007) are particularly helpful to unpack these findings. Putnam’s concept of ‘bonding’ and ‘bridging’ social capital relate to deepening existing relationships and creating new relationships outside existing social circles (Putnam, 2000). Arts engagement facilitated ‘bonding’ relationships by strengthening friendships and sense of collective identity through shared interest, physical synchrony and a common goal and facilitating a more comfortable route to reciprocate peer support around mental health. Some men avoid expressive intimacy or self-disclosure due to a perceived association with femininities and favour masculine interaction styles such as ‘closeness by doing’ (Migliaccio, 2010). Arts engagement offered an interaction style where connectedness and peer support were process by-products of shared activities rather than explicit objectives. This facilitated social connection and a more relaxed environment to organically discuss mental health likely lessened men’s perceptions of their own needs and vulnerabilities while preserving masculine identities. The benefits of this approach have been reported elsewhere, and the current findings illuminate the power arts engagement for normalizing conversations around ordinarily private topics and transgress traditional linkages between masculinities and reticence for self-disclosing mental health challenges (Carroll *et al*., 2014; Seaton *et al*., 2017; Oliffe *et al*., 2020). Bridging relationships were formed with the wider community through the gifting of arts products or public performances. This re-ignited a sense of purpose and usefulness for men which many participants felt they had lost through the displacement of perceived masculine roles. These more outward experiences of arts participation among older men may contrast with older women’s experiences which Liddle *et al*. (Liddle *et al.*, 2013) and Reynolds (Reynolds, 2010) suggest tends to manifest inward experiences of thinking, sensing and feeling.

This also aligns with Atchley’s (Atchley, 1989) continuity theory which notes that adjustment to ageing in life depends on the extent to which an individual can continue to carry out preferred routines, roles, habits and activities. Arts engagement further enhanced self-esteem through a sense of achievement, mastery and routine. The acquisition of new artistic skills and knowledge, resulting in the production of an artistic product or performance aligns with the concept of ‘mastery experience’ in social cognition theory where self-esteem is improved through the successful completion of given tasks (Bandura, 1986). Moreover, gaining a sense of routine resonates with the masculine concepts of discipline and control where men can (re)gain control and order that may have been previously associated with employment (Courtenay, 2004; Robertson *et al*., 2013). Therefore, opportunities should be afforded to men within arts programme to connect by doing, learn new skills and knowledge, produce tangible outputs that can benefit others, and incorporate opportunities for informal and formal feedback to enhance social connectedness and self-esteem.

Finally, emotional regulation may be explained by the concepts of ‘attention deployment’, ‘cognitive change’ and ‘response modulation’ in Gross and Thompson’s (Gross and Thompson, 2007) process model of emotional regulation. With regard to attentional deployment, the arts provided a ‘distraction’ from difficult emotions and situations and complete absorption or ‘concentration’ in a task. Participants spoke of the opportunity to self-reflect and to attach new and deeper meanings to emotions and/or experiences through the art process. This provided men with an alternative outlet to express emotions and to ‘take off the mask’ of emotional restrictiveness associated with masculinities (Connell, 2005) to access a subaltern form of therapeutic catharsis. This resonates with ‘cognitive change’, relating to the appraisal of an experience so as to alter its emotional meaning, and ‘response modulation’, where emotions are released or vented to change previous emotional states (Gross and Thompson, 2007). Therefore, arts engagement can enable men to communicate emotions and thoughts through a self-reflective process which hold unique opportunities for men to attach new meanings to their life experiences and transform masculine identities which has been previously highlighted as critical for the transition to successful ageing among men (Oliffe *et al*., 2013). Further research should explore the utility and sustainability of these theory constructs to interpret and gauge the impact of the arts on men’s mental health through longitudinal qualitative and quantitative research. Exploring the impact of other arts forms is also recommended to ascertain which may be more acceptable among specific sub-groups of men.

Men favoured arts activities that were advertised as being specifically for men, that were delivered in familiar spaces and that were informal and fun. This is consistent with wider literature on the acceptability of health interventions among men (Robertson *et al*., 2013; Seaton, 2017). Moreover, men’s arts engagement was gradual and responded incrementally to exposures to arts activities at men’s shed and mental health recovery groups. Therefore, introducing and integrating arts activities within existing groups can be a strategic approach to build the credibility and legitimacy of men’s arts engagement within group settings. Reframing art arts engagement around men’s interests such as altruism and providing for others—rather than concepts of ‘health’ or ‘creativity’ that may be considered unmasculine—showed promise at overcoming engagement barriers. This finding is consistent with literature highlighting the importance of working with (and not against) cultural ideals of masculinities (Galdas *et al*., 2014) and the benefits of reframing potentially emasculating processes including help-seeking as strength-based actions for regaining control over one’s positive health (Gough and Novikova, 2020). However, in utilizing such an approach, it is crucial to avoid reinforcing such hegemonic masculine ideals that are implicated in men’s poor mental health and that amplify inequities between men and women in all their respective diversities. Therefore, reframing the arts as masculine to engage men is complex. For example, previous research has cautioned against reliance on approaches that embody and sustain traditional masculine ideals (Robertson and Williamson, 2005) and the need to diversify and transform men’s mental health promotion programs to be inclusive of a plurality of masculinities (Gough, 2006). Indeed, there have been calls to incorporate more gender-transformative approaches in men’s health interventions that aim to promote gender equality and healthy masculinities (Paretz *et al*., 2018; Ruane-McAteer *et al*., 2020). The arts offer unique opportunities to garner gender-transformative actions wherein men reflect on, and challenge, hegemonic ideals through artistic expression. That said, reframing the arts around men’s interests at the beginning of a programme and moving towards gender-transformative gains as trust and credibility are garnered is likely the best approach. Previous research has also highlighted that more affluent men often have greater affordances and masculine capital to reshape masculinities along ‘healthier lines’ compared with men experiencing social disadvantage (Gough, 2006). Clearly, more research is needed to distil tailored gender-transformative approaches in men’s arts programmes to effectively respond to a range of mental health inequities. A strong sense of relatedness among men was crucial for bringing about sustained engagement. This is consistent with wider literature which identifies homogeneity of shared social backgrounds as important for safety and sustained engagement (Carroll *et al*., 2014; Galdas *et al*., 2014). Delivering arts programmes to established men’s groups might be a useful starting point to building the locale-specific credibility of programmes in place-based settings. Facilitators delivering arts programmes should adopt strength-based approaches that emphasize the existing strengths, capacities, emotions and virtues of men in order to encourage their potential and promote well-being (Englar-Carlson and Kiselica, 2013). This might include activities and discussions that highlight the strengths and values of men, utilizing existing capacities within the group through peer-mentor approaches and selecting an art form that requires no previous experience or existing skill level to participate. This may also help alleviate concerns among some men of a perceived lack of artistic flare. All participants engaged with active rather than passive participation in arts within their respective groups which resonates with previous men’s health research that highlighted men’s preference for more interactive and action-orientated approaches (Englar-Carlson and Kiselica, 2013). Men favoured arts activities that adopted a modular approach where each session had an end goal that contributed to pre-determined tangible outcomes. This aligns with the hegemonic masculine ideals of logic and rational thinking (Connell and Messerschmidt, 2005) and wider literature highlighting men’s preferences for solution-focussed or problem-solving approaches in health promotion programmes (Galdas *et al*., 2014; Robertson *et al*., 2018). Flexibility with participation and enabling a sense of autonomy and ownership among the group may be operationalized by consulting with men in the development process, incorporating feedback loops and being flexible with the arrangements for each session. These have all been reported as useful engagement strategies in the wider literature (Robertson *et al*., 2013; Seaton *et al.*, 2017; Lefkowich and Richardson, 2018) and specifically in programmes with sustained co-design with men, such as that with male prisoners (Robinson *et al*., 2022). Finally, men noted that facilitators should be encouraging, non-hierarchical, adapt to the needs of the group and lead alongside the group rather than being an authoritative figure. Similar desirable personal qualities in a men’s health facilitator have been reported elsewhere (Carroll *et al*., 2014; Galdas *et al*., 2014; Robertson *et al*., 2018).

In terms of limitations of this work, this study sought to include the voices of a range of low SES men, however, the average age of men in the cohort was 63 years old and they were almost exclusively white British/Irish. Therefore, the findings may not be inclusive or ‘representative’ of all men of low SES in Northern Ireland. While a strength of the study is the in-depth focus on one relatively homogenous group of men, the study could have benefited from recruiting greater numbers of men that were of a younger age, which represented a wider range of ethnicities, and men who participate in the arts in mixed-sex groupings. This study also adopted a narrow definition of the arts and did not consider cultural and everyday participation more generally. Moreover, this study set out to recruit men who were already engaged with the arts and thus does not encapsulate the views of men who chose not to or are unable to participate. Further research with a wider range of men might help to highlight other barriers to male engagement in the arts. This requires the intentional targeting of men through arts programmes that incorporate gender-responsive approaches. Failure to consider gender in mental health promotion work may lead to negative views about the likelihood of engaging men and contribute to self-fulfilling failures attributed to men’s lack of self-interest for well-being (Robertson *et al*., 2018). However, as evidenced in this article, taking time to understand gender in a nuanced way, can help to clarify approaches that facilitate men’s engagement in the arts and potential mechanisms that underpin positive mental health outcomes.

## CONCLUSION

The arts can enhance men’s mental health through opportunities for social connectedness, enhanced self-esteem and emotional regulation. This study adds new knowledge in that it elucidates the gendered dimensions of men experiences highlighting the mental health benefits of the arts among men with low SES and strategies to facilitate their arts engagement. Also added is empirical credibility for using Connell’s masculinities framework to guide the development of gender-responsive arts programmes for men. Indeed, the findings of this study coupled with existing guidelines for incorporating gender-related influences in men’s mental health promotion programmes (Struik *et al*., 2019), can inform such developments. Lastly, given the data for the current study was collected pre-COVID-19 there are also temporal considerations for the findings. Foremost, the post-pandemic lobby and opportunities for being physically back ‘in-person’ seem critical to re-establish in helping men quell some of the isolating effects of COVID-19. Herein, the findings from the current study offer timely and poignant reminders about the value of men being there in person through the arts.
